# Supplementary material for: Breaking translational symmetry via polymer chain overcrowding in molecular bottlebrush crystallization
Source: Nat Commun. 2020 May 1;11:2152. doi: 10.1038/s41467-020-15477-5 (PMC7195396; doi:10.1038/s41467-020-15477-5)
Supplement: Supplementary file 1 — Supplementary Information [file 41467_2020_15477_MOESM1_ESM.pdf]

Supplementary Materials for

Breaking translational symmetry via polymer chain overcrowding in molecular bottlebrush crystallization

. Hao Qi[1†], Xiting Liu[1†], Daniel M. Henn[2†], Shan Mei[1], Mark C. Staub[1], Bin Zhao[2]*, Christopher Y. Li[1]*.

Correspondence to: chrisli@drexel.edu (CYL); bzhao@utk.edu (BZ)

Supplementary Notes

mBBC diameter estimation and size analysis were performed using SEM images. Circles were drawn following the contour of the mBBCs with minimum deformation. The circles diameter was used to estimate mBBC diameter, shown in Supplementary Figure 10.

mBBC cap area estimation. A spherical cap's area S, can be calculated using Equation (1);

$$S = 2\pi R \cdot h \qquad (1)$$

Where R is the spherical radius and h is the cap height.

To estimate the cap area, R was estimated as above described by fitting a circle. The cap height was calculated via equation (2)

$$h = R - \sqrt[2]{R^2 - a^2} \quad (2)$$

Where R is the spherical radius and a is the base radius. a can be measured directly from SEM images, shown in Supplementary Figures 11 (A-C).

Supplementary Figures

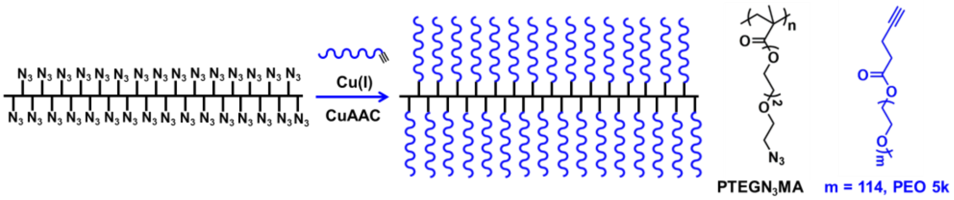

Supplementary Figure 1. A schematic illustration for the synthesis of PEO molecular bottlebrushes by using Cu(I)-catalyzed alkyne-azide cycloaddition reaction to graft alkyne end-functionalized PEO with a molecular weight of 5k Da onto azide-functionalized backbone polymer PTEGN3MA.

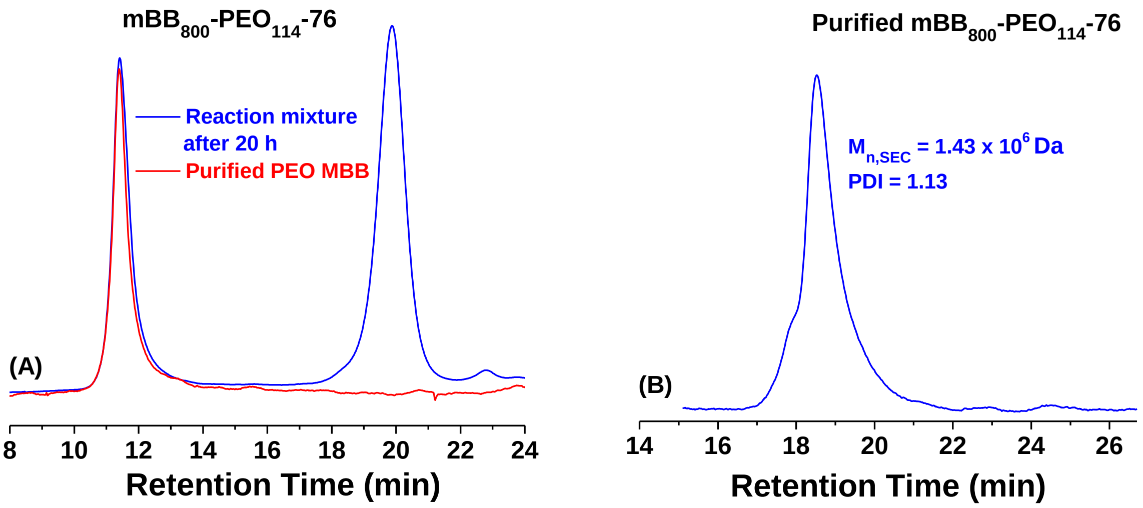

Supplementary Figure 2. (A) SEC traces before and after purification of mBB_{800}-PEO_{114}-76 using PSS GRAL columns. (B) SEC trace of purified mBB_{800}-PEO_{114}-76 using Agilent Mixed-B columns.

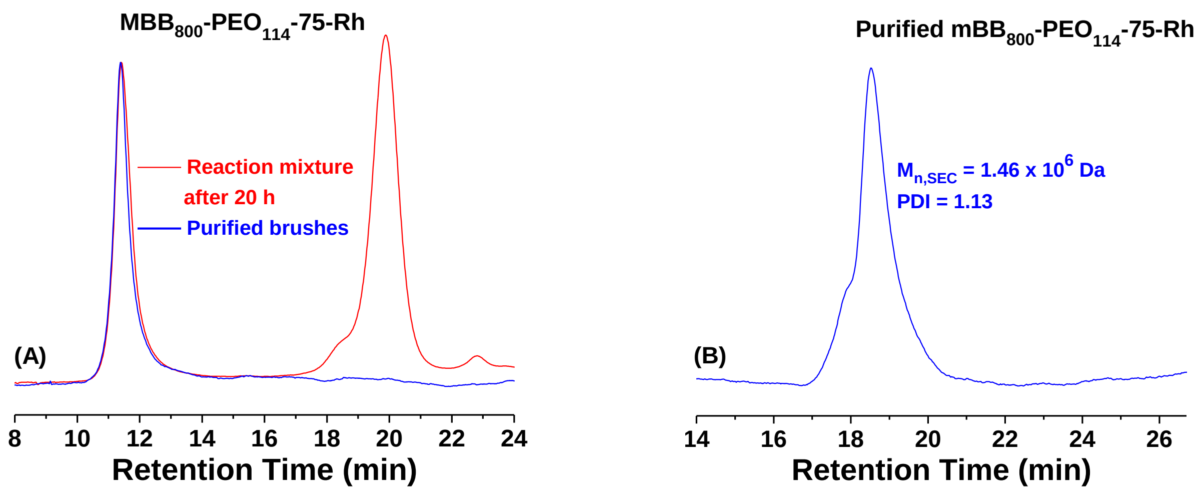

Supplementary Figure 3. (A) SEC traces before and after the purification of mBB_{800}-PEO_{114}-75-Rh using PSS GRAL columns. (B) SEC trace of the purified mBB_{800}-PEO_{114}-75-Rh using Agilent Mixed-B columns.

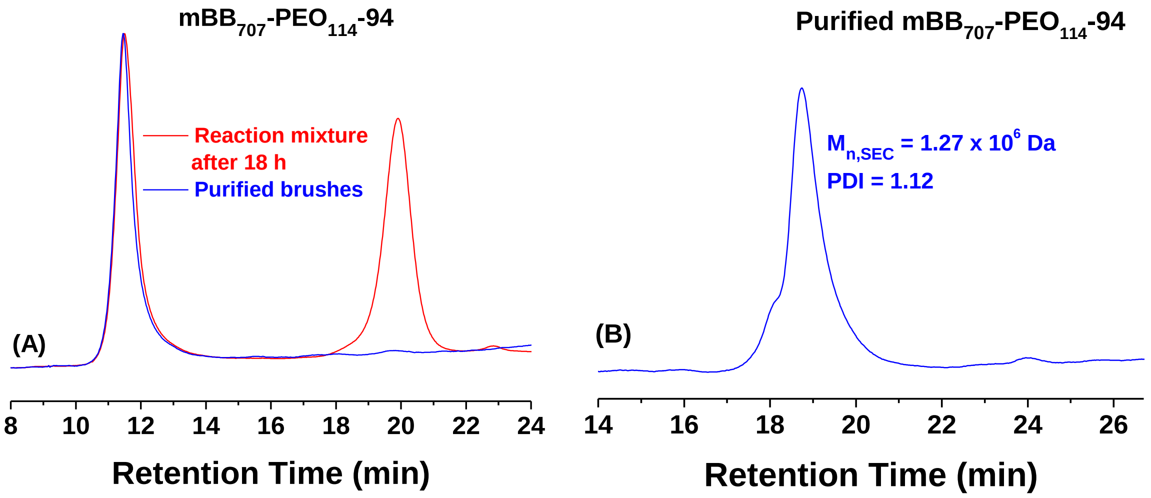

Supplementary Figure 4. (A) SEC traces before and after the purification of mBB_{707}-PEO_{114}-94 using PSS GRAL columns. (B) SEC trace of the purified mBB_{707}-PEO_{114}-94 using Agilent Mixed-B columns.

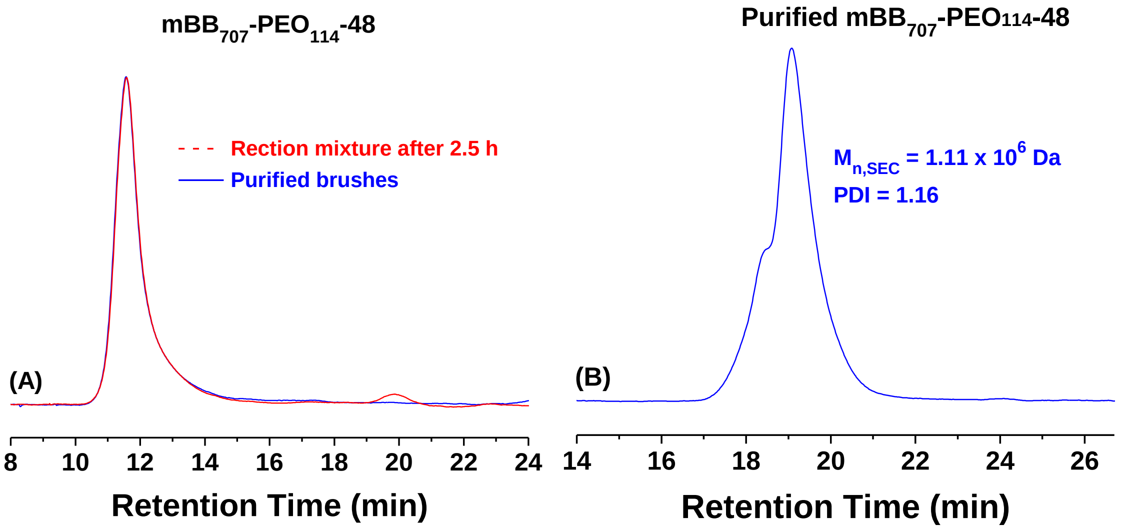

Supplementary Figure 5. (A) SEC traces before and after the purification of mBB_{707}-PEO_{114}-48 using PSS GRAL columns. (B) SEC trace of the purified mBB_{707}-PEO_{114}-48 using Agilent Mixed-B columns.

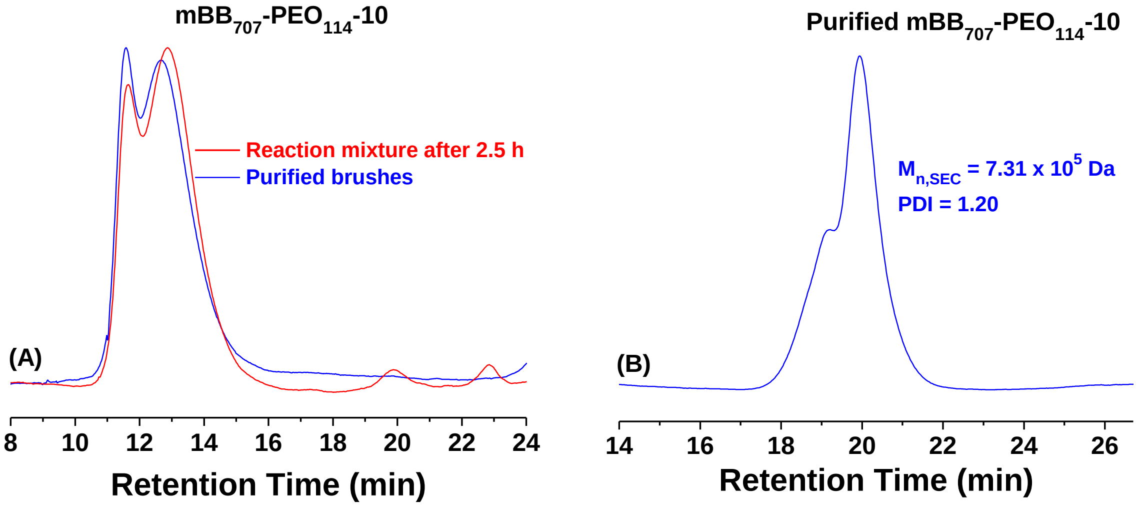

Supplementary Figure 6. (A) SEC traces before and after the purification of mBB$_{707}$-PEO$_{114}$-10 using PSS GRAL columns; the appearance of two peaks was due to the total exclusion of a portion of brush molecules in SEC analysis with PSS GRAL columns. (B) SEC trace of the purified mBB$_{707}$-PEO$_{114}$-10 using Agilent Mixed-B columns.

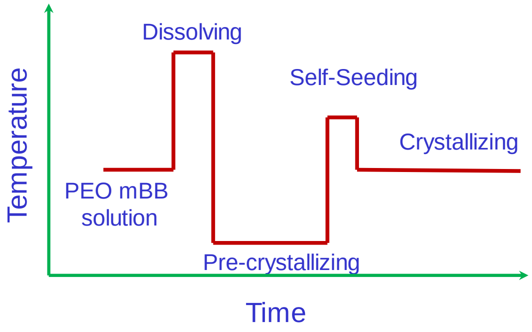

Supplementary Figure 7. Temperature profile of the self-seeding crystallization process.

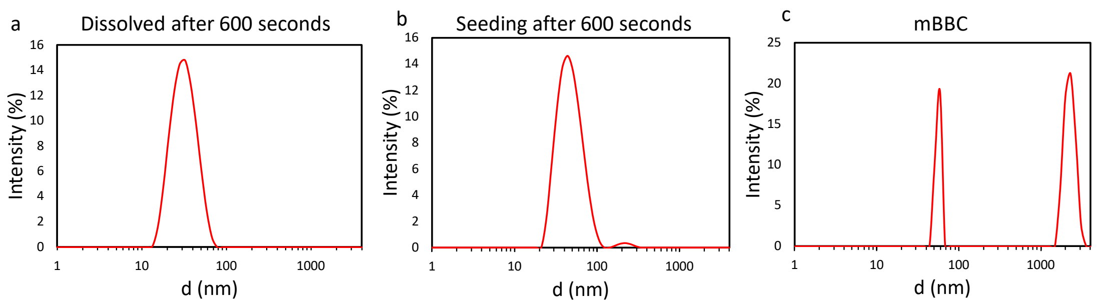

Supplementary Figure 8. Dynamic light scattering of (a) a completely dissolved mBB solution; (b) an mBB seed solution before crystallization and (c) an mBBC suspension after crystallization.

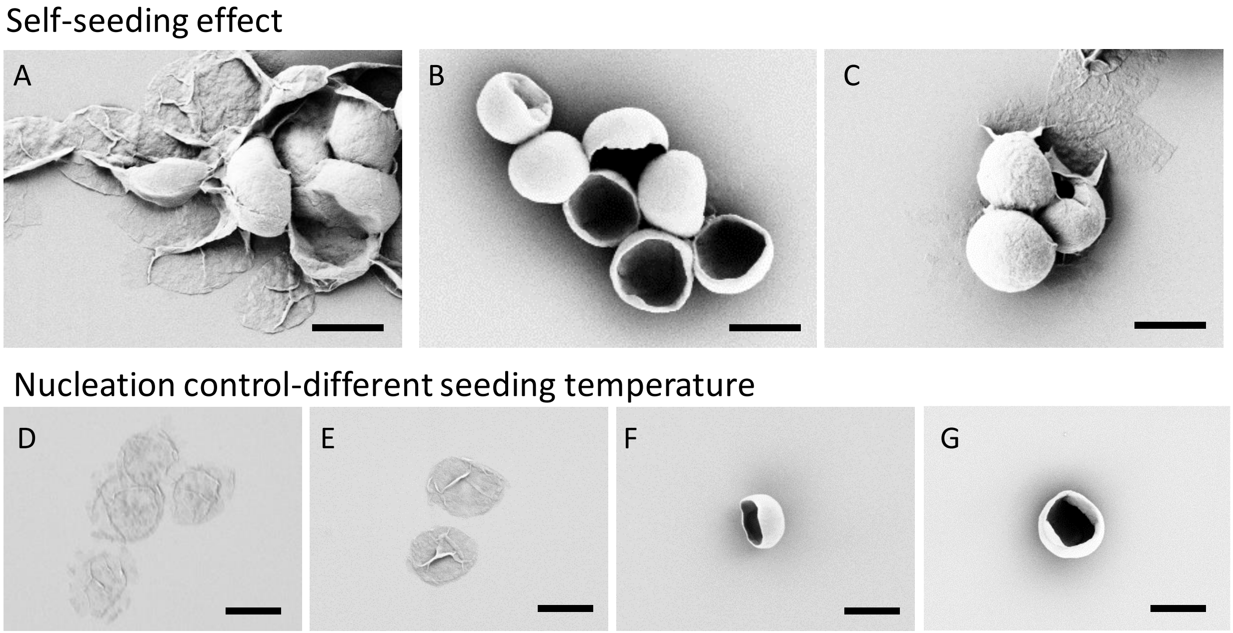

Supplementary Figure 9. Self-seeding solution crystallization of mBBs. (A) to (C) SEM images of typical PEO mBBCs that were crystallized at 20 °C via various routes: (A) directly quenching the dissolved mBB solution, (B) seeding at 44.5°C, and (C) seeding at 45 °C. (D) to (G) SEM images show controlling the size of mBBC opening via tuning of seeding temperature. At the concentration of 0.01 wt. %, the PEO mBB solution was seeded at 44.1 °C (D), 44.3 °C (E), 44.5 °C (F) and 44.8 °C (G). Scale bars are 2 μm in all images. Note that in the crystal growth process, self-seeding is critical to the formation of clear morphology of mBBCs (A-C). (D-G) show that, as the self-seeding temperature slightly increases, the total amount of the seeds in the solution decreases due to continued dissolution of the polymer crystal, which enables an individual cap-like crystal to further develop into an mBBC with an increasingly smaller opening.

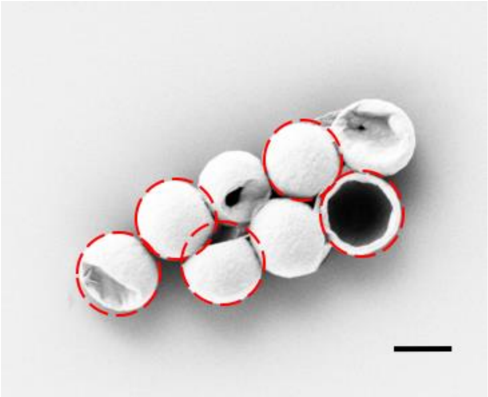

Supplementary Figure 10. SEM image of mBB_{707}-PEO_{114}-48 mBBC showing the size estimation analysis. Scale bar: 2 µm.

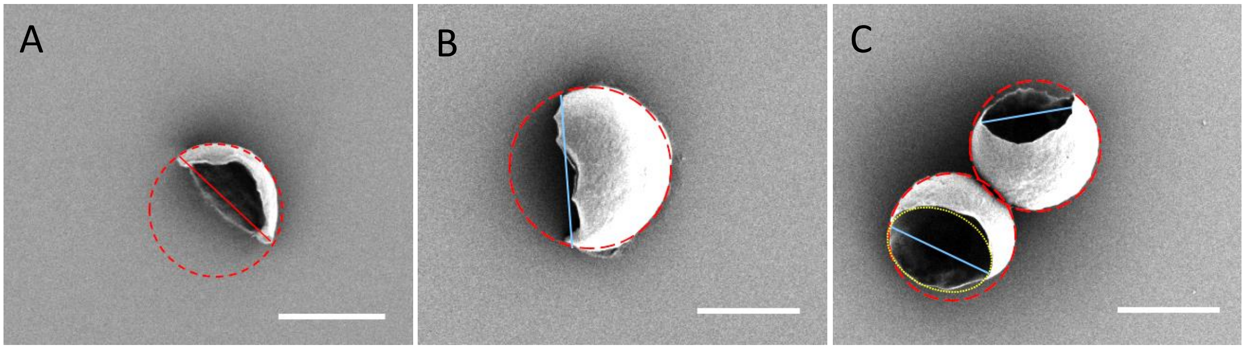

Supplementary Figure 11. SEM images of mBB_{800}-PEO_{114}-76 mBBC showing the *R* and *a* estimation analysis at different crystallization time. (A) 10 min; (B) 20 min and (C) 30 min. Scale bars are 2 μm.

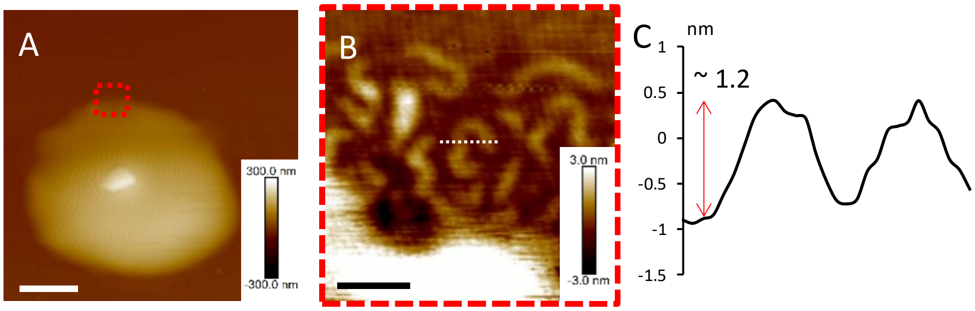

Supplementary Figure 12. AFM images of an mBBC after ultrasonication (A-B) and corresponding height profile (C). (B) displays the enlarged image of the dotted line squared area in (A). Individual mBB molecules can be seen at the edge of the mBBC, showing the connection of the mBB molecule and the crystal. Scale bar 1µm (left) and 100nm (right).

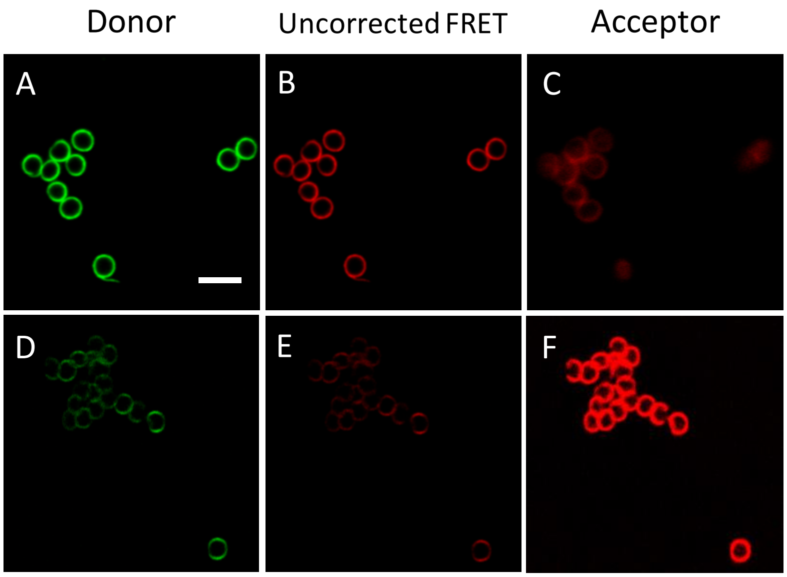

Supplementary Figure 13. Confocal fluorescence images in FRET experiments for open (A-C) and closed (D-F) crystalsomes. The green color represents the NBDA emission and the red color represents Rhodamine B emission. A 488-nm laser was used to excite NBDA (A, B, D, E) and a 543 nm laser for Rhodamine B (C, F). Open (A) and closed (D) mBB_{800}-PEO_{114}-75-Rh mBBCs were treated with NBDA and the emission of NBDA was recorded. Uncorrected FRET images (B, E) were acquired with 488-nm excitation and 600-660 nm filter. Emission of Rhodamine B was recorded, where (C) is the opened mBB_{800}-PEO_{114}-75-Rh mBBCs and (F) is the closed mBB_{800}-PEO_{114}-75-Rh mBBCs obtained with 543 nm excitation. (Scale bar: 5 um)

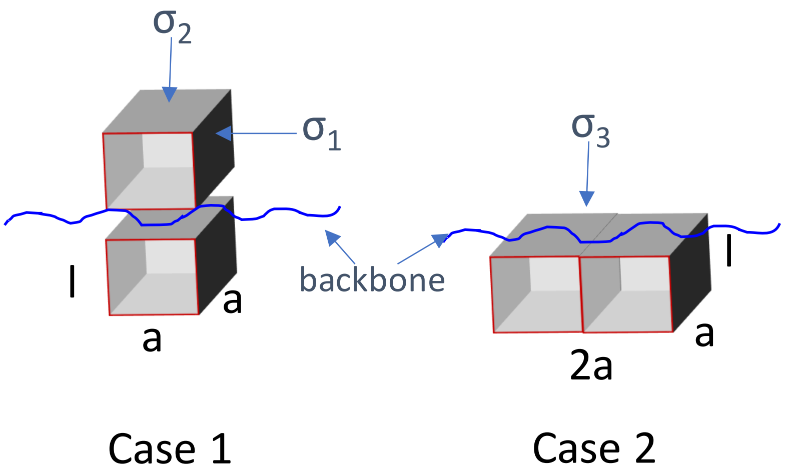

Supplementary Figure 14. Total free energy of the mBB upon crystallization in two cases. In case 1, the PEO side chains form crystals on both sides of the backbone while in case 2, the side chains form crystals on one side of the backbone. Assuming the crystal thickness is l and the lateral dimension is a. σ_1 , σ_2 , σ_3 are the surface free energies of lateral surface, top surface, and the surface in contact with backbone.

For case 1, we have $\Delta G = 8la\sigma_1 + 2a^2\sigma_2 + 2a^2\sigma_3 - \Delta g$ (3)

For case 2, we have $\Delta G = 6la\sigma_1 + 2a^2\sigma_2 + 2a^2\sigma_3 - \Delta g$ (4)

ΔG is the free energy of the crystal, and Δg is the bulk free energy of fusion. For simplicity, we assumed the two lateral sides have the same dimension a. From the above two equations, case 2 has a lower free energy by $2la\sigma_1$. The asymmetry nature was also confirmed by the FRET results.
