# Supplementary material for: Breaking translational symmetry via polymer chain overcrowding in molecular bottlebrush crystallization
Source: Nat Commun. 2020 May 1;11:2152. doi: 10.1038/s41467-020-15477-5 (PMC7195396; doi:10.1038/s41467-020-15477-5)
Supplement: Supplementary file 3 — Reporting Summary [file 41467_2020_15477_MOESM3_ESM.pdf]

nature research

Corresponding author(s): Christopher Li

- [ ] Initial submission
- [x] Revised version
- [ ] Final submission

Editorial Policy Checklist

This form is used to ensure compliance with Nature Research editorial policies related to research ethics and reproducibility. For further information, please see our Authors & Referees site. All relevant questions on the form must be answered.

Competing interests

Policy information about competing interests

Competing interests declaration

In the interest of transparency and to help readers form their own judgements of potential bias, Nature Research journals require authors to declare any competing financial and/or non-financial interest in relation to the work described in the submitted manuscript.

- [x] No, I declare that the authors have no competing financial or non-financial interests as defined by Nature Research.
- [ ] Yes, I declare that the authors have a competing interest as defined by Nature Research

Data availability

Policy information about availability of data

Data availability statement

All manuscripts must include a data availability statement. This statement should provide the following information, where applicable:

- Accession codes, unique identifiers, or web links for publicly available datasets
- A list of figures that have associated raw data
- A description of any restrictions on data availability

- [x] A full data availability statement is included in the manuscript.

Mandated accession codes (where applicable)

Confirm that all relevant data are deposited into a public repository and that accession codes are provided.

- [ ] All relevant accession codes are provided
- [ ] Accession codes will be available before publication
- [x] No data with mandated deposition

Data presentation

Image integrity

- [x] Confirm that all images comply with our image integrity policy.

Unprocessed data must be provided upon request. Please double-check figure assembly to ensure that all panels are accurate (e.g. all labels are correct, no inadvertent duplications have occurred during preparation, etc.).

Data distribution

Present data in a format that shows data distribution (dot-plots or box-and-whisker plots).
Define all box-plot elements (e.g. center line, median; box limits, upper and lower quartiles; whiskers, 1.5x interquartile range; points, outliers).
If using bar graphs, overlay the corresponding dot plots.

- [x] Confirm that all data presentation meets these requirements and that individual data points are shown.

Specific policy considerations

Some types of research require additional policy disclosures. Please indicate whether these apply to your study. If you are not certain, please read the appropriate section before selecting a response.

Does not apply	Involved in the study	
x		Custom software or computer code
x		Macromolecular structural data
x		Research animals and/or animal-derived materials that require ethical approval
x		Human research participants
x		Clinical data

I certify that all the above information is complete and correct.

Typed signature: Christopher Li

Date: Mar 2, 2020

This checklist template is licensed under a Creative Commons Attribution 4.0 International License, which permits use, sharing, adaptation, distribution and reproduction in any medium or format, as long as you give appropriate credit to the original author(s) and the source, provide a link to the Creative Commons license, and indicate if changes were made. The images or other third party material in this article are included in the article's Creative Commons license, unless indicated otherwise in a credit line to the material. If material is not included in the article's Creative Commons license and your intended use is not permitted by statutory regulation or exceeds the permitted use, you will need to obtain permission directly from the copyright holder. To view a copy of this license, visit http://creativecommons.org/licenses/by/4.0/
